# Preterm or Not – An Evaluation of Estimates of Gestational Age in a Cohort of Women from Rural Papua New Guinea

**DOI:** 10.1371/journal.pone.0124286

**Published:** 2015-05-06

**Authors:** Stephan Karl, Connie S. N. Li Wai Suen, Holger W. Unger, Maria Ome-Kaius, Glen Mola, Lisa White, Regina A. Wangnapi, Stephen J. Rogerson, Ivo Mueller

**Affiliations:** 1 Walter and Eliza Hall Institute of Medical Research (WEHI), Melbourne, Australia; 2 Department of Medical Biology, The University of Melbourne, Melbourne, Australia; 3 Department of Medicine (Royal Melbourne Hospital), The University of Melbourne, Melbourne, Australia; 4 Papua New Guinea Institute of Medical Research (PNG IMR), Goroka, Papua New Guinea; 5 Department of Obstetrics and Gynaecology, University of Papua New Guinea, Port Moresby, Papua New Guinea; 6 Centre for Tropical Medicine, Nuffield Department of Clinical Medicine, University of Oxford, Oxford, United Kingdom; 7 Mahidol-Oxford Tropical Medicine Research Unit, Faculty of Tropical Medicine, Mahidol University, Bangkok, Thailand; 8 Barcelona Institute for Global Health (ISGlobal), Barcelona, Spain; Instituto de Ciências Biomédicas / Universidade de São Paulo - USP, BRAZIL

## Abstract

**Background:**

Knowledge of accurate gestational age is required for comprehensive pregnancy care and is an essential component of research evaluating causes of preterm birth. In industrialised countries gestational age is determined with the help of fetal biometry in early pregnancy. Lack of ultrasound and late presentation to antenatal clinic limits this practice in low-resource settings. Instead, clinical estimators of gestational age are used, but their accuracy remains a matter of debate.

**Methods:**

In a cohort of 688 singleton pregnancies from rural Papua New Guinea, delivery gestational age was calculated from Ballard score, last menstrual period, symphysis-pubis fundal height at first visit and quickening as well as mid- and late pregnancy fetal biometry. Published models using sequential fundal height measurements and corrected last menstrual period to estimate gestational age were also tested. Novel linear models that combined clinical measurements for gestational age estimation were developed. Predictions were compared with the reference early pregnancy ultrasound (<25 gestational weeks) using correlation, regression and Bland-Altman analyses and ranked for their capability to predict preterm birth using the harmonic mean of recall and precision (F-measure).

**Results:**

Average bias between reference ultrasound and clinical methods ranged from 0–11 days (95% confidence levels: 14–42 days). Preterm birth was best predicted by mid-pregnancy ultrasound (F-measure: 0.72), and neuromuscular Ballard score provided the least reliable preterm birth prediction (F-measure: 0.17). The best clinical methods to predict gestational age and preterm birth were last menstrual period and fundal height (F-measures 0.35). A linear model combining both measures improved prediction of preterm birth (F-measure: 0.58).

**Conclusions:**

Estimation of gestational age without ultrasound is prone to significant error. In the absence of ultrasound facilities, last menstrual period and fundal height are among the more reliable clinical measures. This study underlines the importance of strengthening ultrasound facilities and developing novel ways to estimate gestational age.

## Introduction

Knowledge of gestational age (GA) is a prerequisite for the provision of optimal care to mother, fetus and neonate. Examples include the monitoring of maternal weight gain through the course of the pregnancy [1], the administration of steroids in women with suspected pre-term labour [2], ultrasound detection of suboptimal fetal growth, as well as intensified observation and management of preterm newborns (preterm birth [PTB], < 37 weeks gestation). Additionally, precise estimates of GA are required to identify causes of, and evaluate interventions to prevent, PTB and fetal growth restriction (FGR) and their respective contribution to the high burden of low birthweight (< 2,500g) in low-resource settings [3]. Low birthweight is associated with maternal undernutrition and malaria; increases infant mortality rates and predisposes to ill health in adult life [4,5].

In industrialised countries GA is usually estimated with the help of fetal biometric measurements taken in early pregnancy [6]. Ultrasound-predicted GA according to fetal crown-rump length (head circumference or femur length in early second trimester) is used to corroborate estimated delivery dates as per last menstrual period (LMP), and in cases of absent LMP (unknown, highly irregular menstrual cycles) or significant disagreement, GA is estimated by first trimester ultrasound alone [6]. In low-resource environments high-quality fetal biometric measurements can be obtained by locally trained health workers and the acceptability of ultrasound appears to be good [7–10]. However, ultrasound machines and training are costly, and may not be a priority in resource-constrained countries with fragile health care systems. This, together with late presentation to antenatal clinic, currently precludes widespread use of sonographic early pregnancy dating in these settings [11,12]. Instead, health workers rely on other means of estimating GA, particularly when operating in poorly-resourced rural areas. Available alternatives include LMP, symphysis pubis-fundal height (SFH) (single or multiple measurements) [13,14], quickening, neonatal physical and neurological maturity assessments (Dubowitz or Ballard score [BS]) [15,16], and mid- and late pregnancy fetal biometry [7]. Their accuracy to predict gestational age at delivery may be suboptimal [17].

Papua New Guinea (PNG) is a developing country in the South Pacific with a largely rural population and high maternal and infant mortality rates [18,19]. Ultrasound is a scarce commodity in the public sector [20], and late presentation to antenatal clinic is a frequent occurrence [21,22]. Little is known about the precision and usefulness of clinical estimators of GA in PNG despite their frequent use [23].

We compared the performance of established alternative estimators of GA in a cohort of Melanesian women from rural PNG with fetal biometry in the first half of pregnancy and assessed whether combination of various measures in mathematical models could improve GA estimation.

## Materials and Methods

### Study location

Data collection for this research was conducted between November 2009 and December 2012 at eight health facilities in the Madang municipality on the North coast of PNG. The burden of low birthweight in the study area is high [24–27], and pregnancy care is largely provided by government or church-based health centres with no or limited access to ultrasound.

### Study design

Data were collected as part of a randomised controlled trial investigating the impact of intermittent preventive treatment in pregnancy with azithromycin-containing regimens to reduce low birthweight (NCT01136850) [26]. The present study assessed the performance of different established clinical measures (individually or in combination) to determine GA and detect PTB, using early pregnancy fetal biometry as the reference method for pregnancy dating. Furthermore we evaluated the combination of measures in mathematical models.

**Fig 1:**
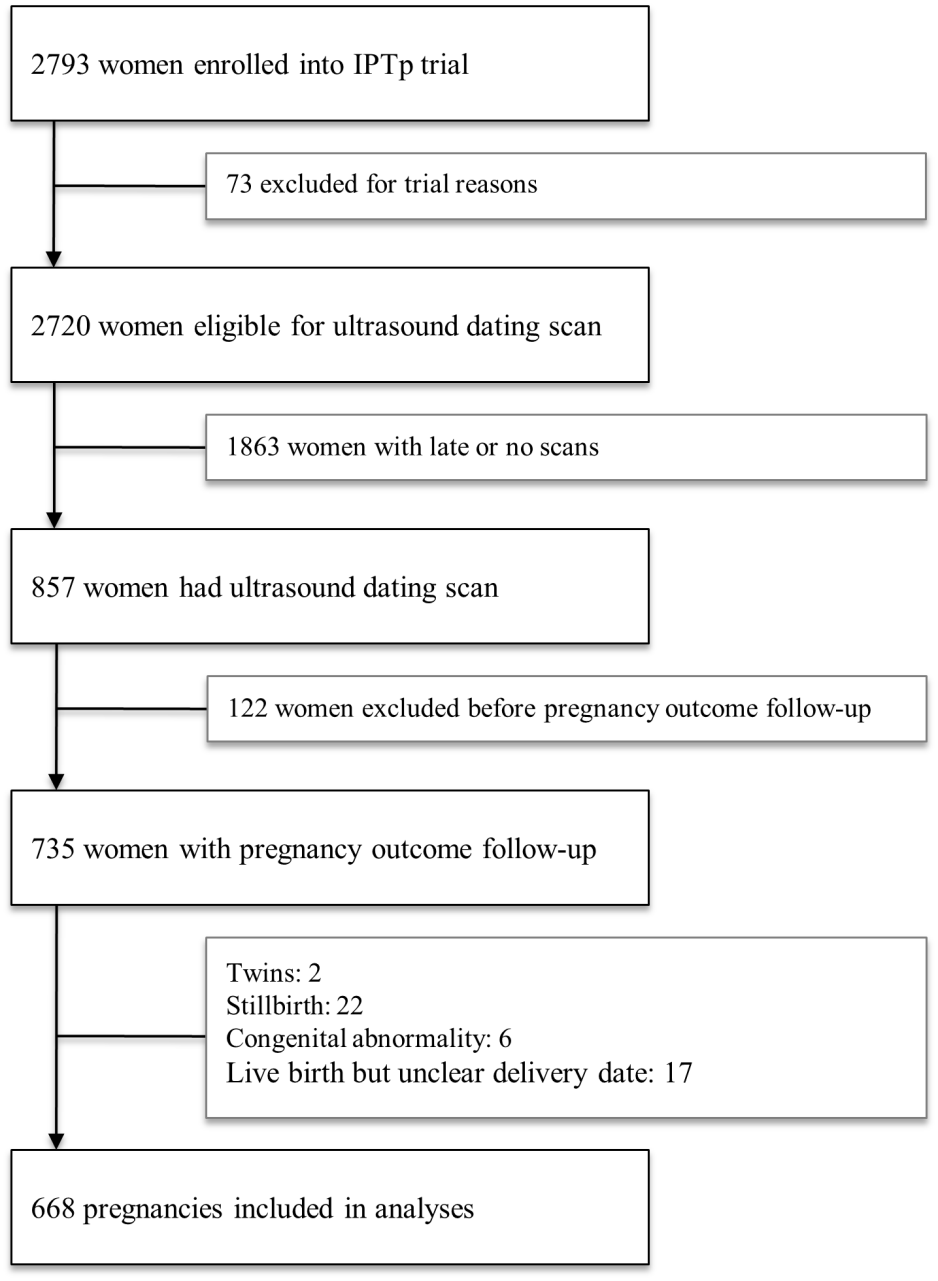
Participant Flowchart.

Women enrolled in the parent trial (age 16–49 years, singleton pregnancy, no co-morbidities, SFH ≤26 cm) were offered an obstetric ultrasound scan within a week of enrolment and were included in the present evaluation if they were <25 weeks gestation according to fetal biometry. Socio-demographic characteristics were evaluated and a clinical examination was performed at the enrolment visit. Participants were provided with insecticide-treated bed nets and trial interventions. Women were scheduled for two further antenatal study visits and followed until delivery. Birthweights were recorded using electronic infant scales (Cupid 1, Charder Medical, Taiwan; precision: 10 g). Pregnancies complicated by miscarriage, stillbirth, congenital abnormality or events resulting in withdrawal from the parent trial were excluded from this analysis (Fig 1). Research nurses were masked to delivery GAs assigned by each method.

### Gestational age estimation

Reference pregnancy dating was performed according to British Medical Ultrasound Society guidelines using crown-rump length at 6–13 gestational weeks, or head circumference (femur length if unavailable [n = 14]) at 13–25 gestational weeks to estimate GA [6]. A subset of women underwent mid-pregnancy (25w0d [175 d] to 29w6d [209 d]) and late-pregnancy scans (30w0d [210 d] to 35w6d [252 d]): here GA was estimated as per Hadlock *et al*., using a combination of head circumference, abdominal circumference, femur length and biparietal diameter measurements [28]. Study clinicians trained in obstetric ultrasound (MO, HWU) took biometric measurements using a portable scanner (Logiqbook XP, General Electric Medical Systems, UK). Ten percent of ultrasound image stills were randomly selected for external quality control (Dr J Walker, Royal Infirmary of Edinburgh, United Kingdom) and 92.5% of images fulfilled the quality criteria (images that did not pass quality control were excluded from all analyses) [6]. Inter-observer variability was evaluated in ten fetuses, and issues regarding measurement precision were addressed.

Clinical measures to predict GA (collected by a total of 27 research nurses) are summarised in Table 1. The measurements included SFH [13], LMP, quickening and postnatal maturational assessment using BS [16]. Nurses underwent biannual training sessions led by research clinicians to ensure collection of high-quality data. Training used pictorial guides based on the work by Ballard et al [16] and produced by the Malaria in Pregnancy Consortium. Each theoretical training session was followed by supervised maturational assessments on newborns not included in the present study. Areas requiring improvement were highlighted and further individual training provided as necessary. There was no external quality control of BS assessments.

**Table 1:** Abbreviations for Clinical Measures Used the Text.

Method	Abbreviation
Ballard Score (external)	*BS(e)*
Ballard Score (neuromuscular)	*BS(n)*
Ballard Score (total)	*BS(t)*
Last Menstrual Period	*LMP*
Symphysis pubis fundal-height (linear model) [13]	*SFH (linear)*
Symphysis pubis fundal-height (sequential model) [13]	*SFH (sequential)*
Corrected last menstrual period based on PNG guidelines [30]	LMP*

**Note:** LMP* is the corrected last menstrual period based on PNG guidelines.

BS were included in analyses if measured within 96 hours of delivery [16], and were assessed as total, external and neuromuscular BS, according to established methodology [16,29]. GA in days from BS was estimated using Eq 1:

$$BS(days)=7\times \frac{\left(2\times BS+120\right)}{5}$$ (1)

GA by LMP (defined as the first day of the last menstrual bleed, relying upon recall of the women) was calculated assuming a regular 28-day cycle for all women (cycle characteristics data was not collected). Quickening was defined as the date the mother started feeling fetal movements, and information was collected for a subset of women.

SFH was defined as the distance between the upper border of the symphysis pubis (palpated with right index and middle finger) and the uterine fundus (palpated with the lateral aspect of the assessor’s left hand), and measured at enrolment and at two subsequent study visits. Prior to examination, women were asked to empty their bladder. Once a woman had assumed a supine position, SFH was measured (to the nearest cm) using a standard soft tape measure. To avoid observer bias, initial placement of the measuring tape purposely occluded view of the scale by inverting the tape and the scale was only revealed once the SFH had been palpated.

We assessed the performance of two published models estimating GA at delivery from SFH measurements (for details please refer to [13]). The first model is a linear model based on a single SFH measurement taken at first antenatal visit. The second model uses sequential SFH measurements. This model was developed in a study that collected a large number of SFH measurements during each individual pregnancy, estimating GA using all possible triplet combinations between these SFH values. Since in our study a maximum of three SFH measurements were collected per pregnancy, only one such combination (i.e. SFH1, SFH2, SFH3) could be calculated [13]: analysis was restricted to SFHs measured ≥14 days apart.

Furthermore, we assessed the performance of a clinical algorithm that is currently recommended for use in PNG when ultrasound is unavailable (LMP*) [26]. The algorithm proposes correction of LMP-based GA estimates if found > 3 weeks different from SFH, at which point GA is estimated according to SFH and quickening [26]. This analysis was restricted to women with an SFH at first antenatal visit in the range of 20–35 cm (SFH is assumed to equal GA in gestational weeks), as only a small number of women had SFH measurements below this range. Since quickening data was not available for all women, values were imputed based on the assumption that primigravidae and multigravidae start feeling fetal movements at 20 and 18 weeks gestation, respectively, as per PNG guidelines. [30]

Lastly, we evaluated the performance of multiple linear regression models combining the established GA estimates in order to assess whether PTB prediction could be improved.

### Data analysis

Data were double-entered into the trial database (FoxPro 9.0, Microsoft, USA) and analyses were performed using STATA 12.0 (StataCorp, College Station, TX, USA), Mathematica 9.0 (Wolfram Research, Champaign, IL, USA), R 3.1.1 [31], Microsoft Excel and GraphPad Prism 6.0 (GraphPad Inc, La Jolla, CA, USA). A sample size calculation was performed for the parent trial but not for the present study.

Bland-Altman analyses (for mean bias and 95% confidence levels of agreement [LOA]), orthogonal regression (for regression coefficients), intraclass correlation, and Lin’s concordance analyses were used to assess correlation [32,33]. Note that an average bias close to 0 indicates better accuracy and narrow LOA correspond to more precise measurements. The intraclass and concordance correlation coefficients are measures of reliability and reproducibility between methods with higher coefficients indicating better agreement (values <0.3 are usually regarded as low, 0.3–0.7 as moderate and >0.7 as strong correlation). Sensitivity, specificity and predictive values of each method to predict PTB were calculated following two-way tabulation and the performance of methods was ranked based on their location in the receiver operating characteristic space using F-measures (F-harmonic means of sensitivity and positive predictive value and a surrogate for the area under the receiver operating characteristic curve).

In addition, six multiple linear regression models with different combinations of clinical measures as covariates were fitted to predict GA at delivery. The multiple linear regression model with the best predictive accuracy was selected according to *k*-fold cross-validation and the F-measure.

Other analyses included assessments of the potential impact of the timing and assessor of BS on GA estimation precision as well as exclusion of outliers from LMP analyses.

### Ethics

All women provided written informed consent at recruitment. The study was approved by the PNG Institute of Medical Research Institutional Review Board (0815), the PNG Medical Research Advisory Council (08.01) and the Melbourne Health Human Research Ethics Committee (2008.162). Data used in this study were routinely collected as part of the trial protocol.

## Results

Of 2,793 women enrolled in the parent trial, 857 had a reference USS (i.e., scan before 24 weeks). Of these women, 735 had a complete pregnancy outcome follow-up (Fig 1). Twenty-two women (3.0%) had a miscarriage or stillbirth, six (0.8%) had a newborn with a congenital abnormality, two were twin pregnancies, and a further 17 were excluded as the exact date of delivery was unknown, leaving a final cohort of 688 women for analysis. Half of the women were primigravid, two-thirds resided in rural areas and the majority was literate (Table 2).

**Table 2:** Enrolment Characteristics of Pregnant Women (n = 688) from Rural Madang Province, Papua New Guinea, 2009–2012.

Characteristic		Mean ± SD or % (n)
Age (years)		24.3 ± 5.4
Mid-upper arm circumference (cm)		24.0 ± 2.8
Body mass index (kg/m^2^)		22.7 ± 3.1
Height (cm)		153.9 ± 5.9
Literate (≥3 years of formal schooling)		90.7 (624)
Gravidity		
	1	52.3 (360)
	2	22.1(152)
	>3	25.6 (176)
Anaemia (< 70 g/L)		6.5 (45)
Malaria*		14.8 (102)
Smoker		17.6 (121)
Rural residence		58.2 (399)
Maternal ethnic grouping		
Madang/Morobe		14.7 (101)
Sepik		53.6 (369)
Highlands		8.1 (56)
New Guinea islands		4.2 (29)
Mixed PNG		19.3 (133)

**Note:** SD, standard deviation.

*by light microscopy and/or qPCR

Mean GA at enrolment by reference USS was 136 days (SD 27; range 39–174), and mean GA at delivery was 275 days (SD 12; range 179–306). Birthweights were available for 660 of 688 infants (95.9%): the mean birthweight was 2,927 g (SD 484; range 900–4,250) and 45.5% (299/657) were male newborns. The prevalence of low birthweight was 15.5% (102/660). Only 2.9% (20/688) of pregnancies were dated using crown-rump length due to late first attendance at antenatal clinic.

### Agreement between established methods

**Fig 2:**
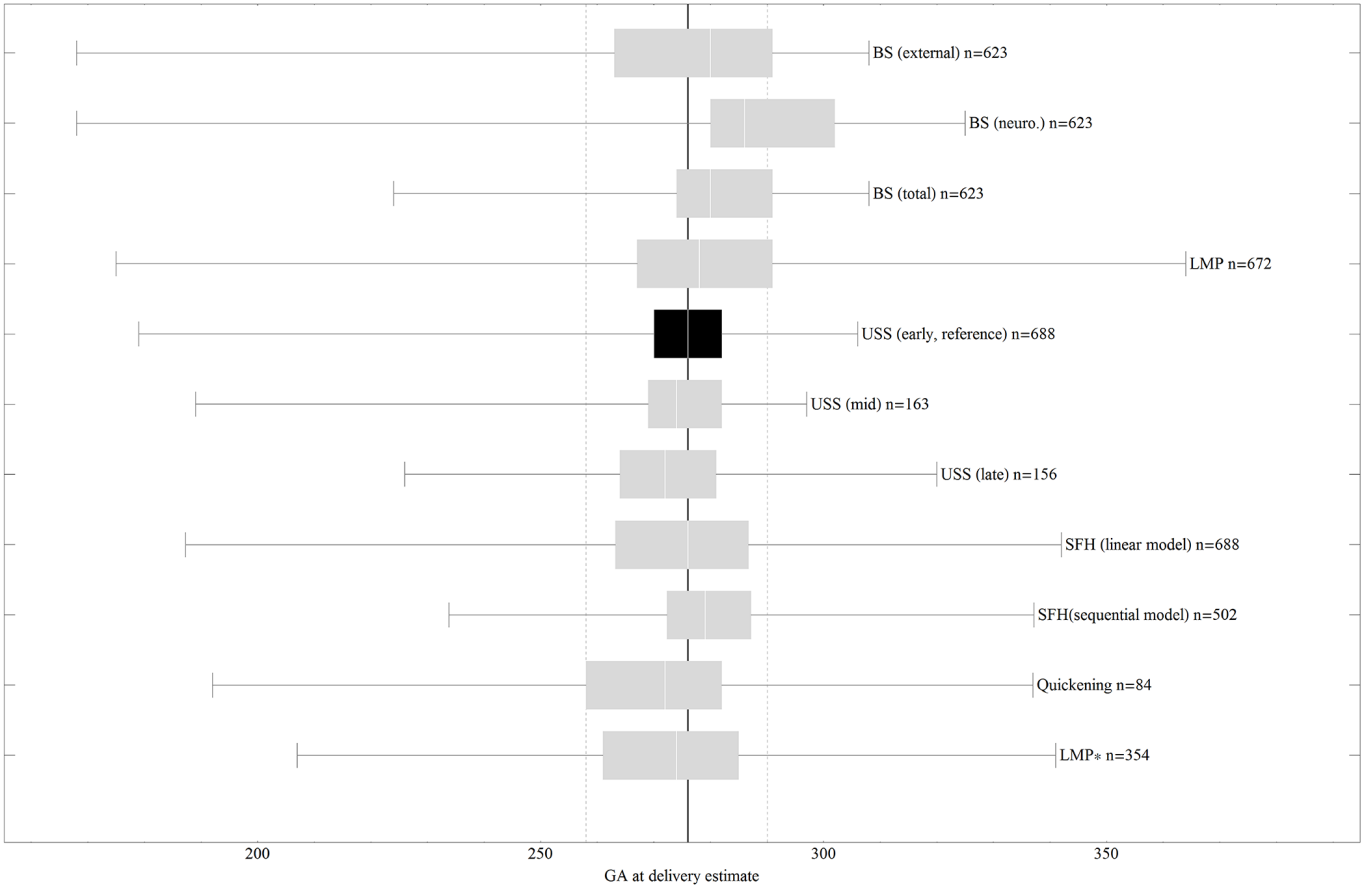
Box-and-Whisker Charts of Estimated GA at Delivery by Method. The continuous bold line denotes the median of the reference and dashed lines denote 5% and 95% centiles of the reference.

The distribution of GA estimates by reference USS in comparison to the other evaluated methods is given in Fig 2 (and, in more detail, in S1 Fig).

**Fig 3:**
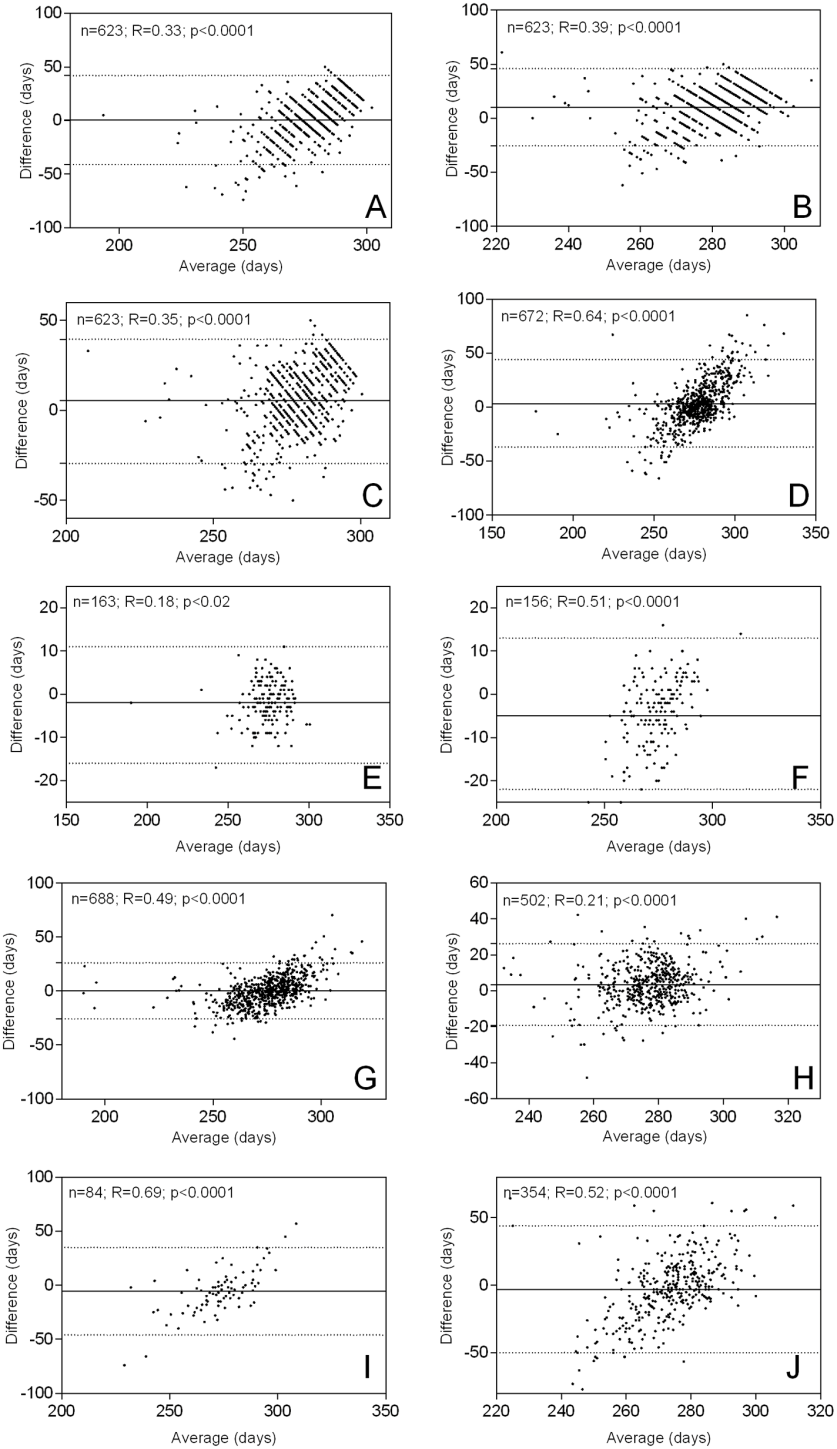
Bland-Altman Plots and Levels of Agreement. A) BS (external); B) BS (neuromuscular); C) BS (total); D) LMP; E) mid-pregnancy ultrasound; F) late-pregnancy ultrasound; G) linear SFH model; H) sequential SFH model; I) Quickening; J) corrected LMP*. The continuous horizontal lines are average levels of agreement. The dashed lines denote the 95% levels of agreement between the clinical estimators and the reference method. R represents the Pearson correlation coefficient and p values indicate significance of the parametric correlations. Significant trends are present in all comparisons, indicating significant variability in the bias across the data range. The correlations are all positive, meaning that the clinical estimators tend to further underestimate lower estimates of GA, which is demonstrated by the high number of PTB predicted by most clinical methods (Table 4).

Table 3 summarises the correlation statistics for GA at delivery in days (mean bias, intraclass correlation and concordance correlation), and Fig 3 shows the corresponding Bland-Altman plots. Correlation plots and best fit curves of orthogonal regression analyses are provided in S2 Fig

**Table 3:** Comparison of Clinical and Late Ultrasound Estimates of Gestational Age Against Reference Ultrasound.

Method	Average bias (95% LOA)	ICC (SE)	Concordance(SE)
Ballard Score (external)	**1** (-41, 42)	**0.11** (0.05)	**0.21** (0.03)
Ballard Score (neuromuscular)	**11** (-25, 46)	**0.09** (0.05)	**0.13** (0.03)
Ballard Score (total)	**6** (-27, 39)	**0.19** (0.06)	**0.22** (0.03)
Last Menstrual Period (LMP)	**3** (-37, 44)	**0.48** (0.05)	**0.38** (0.03)
Scan between 25 & 29 GW	**-2** (-16, 11)	**0.85** (0.04)	**0.85** (0.02)
Scan between 30 & 35 GW	**-5** (-22, 13)	**0.58** (0.08)	**0.66** (0.04)
SFH (linear model)	**0** (-26, 26)	**0.59** (0.09)	**0.64** (0.02)
SFH (sequential model)	**4** (-19, 26)	**0.57** (0.22)	**0.52** (0.03)
Quickening	**-6** (-46, 35)	**0.43** (0.15)	**0.36** (0.07)
LMP*	**-3** (-36, 30)	**0.57** (0.06)	**0.50** (0.03)

**Note:** LOA, 95% confidence levels of agreement (in days); SE, standard error; GW, gestational weeks; LMP, last menstrual period; LMP*, corrected last menstrual period based on PNG guidelines; SFH, symphysis-pubis fundal height; ICC, Intraclass correlation.

Mid- and late-pregnancy USS tended to be associated with increasing discordance to the reference method, however mid-pregnancy scans still resulted in reasonably good agreement with the reference. Agreement between clinical estimates and the reference varied, with intraclass and concordance correlation coefficients ranging from 0.09 to 0.59 and 0.13 to 0.64 respectively. The average bias was generally low (mostly less than ±6 days, Table 3). Overall, BS estimates correlated least well with the reference estimates (Table 3, ICC: 0.09–0.19 and concordance: 0.13–0.22), and the established SFH models, LMP and LMP* correlated better, with narrower levels of agreement (Table 3, ICC: 0.48–0.59; Concordance: 0.38–0.64).

In almost all Bland-Altman analyses we observed statistically significant (Pearson correlation) positive associations between the differences and the averages of the paired measurements (Fig 3). Therefore, the clinical estimates showed a tendency, which was often strong, to further underestimate lower estimates of GA. S1 Table shows linear regression coefficients for average GA vs. difference in GA as determined by each clinical estimator against the reference method (i.e., a linear regression performed on the Bland-Altman data). Based on this regression it should be possible to further correct clinical estimates of GA by linear transformation; however, further studies and extensive comparisons with other datasets would be required to determine whether such a correction would be justified and produce reliable estimates across populations.

### Performance of methods to predict PTB

**Fig 4:**
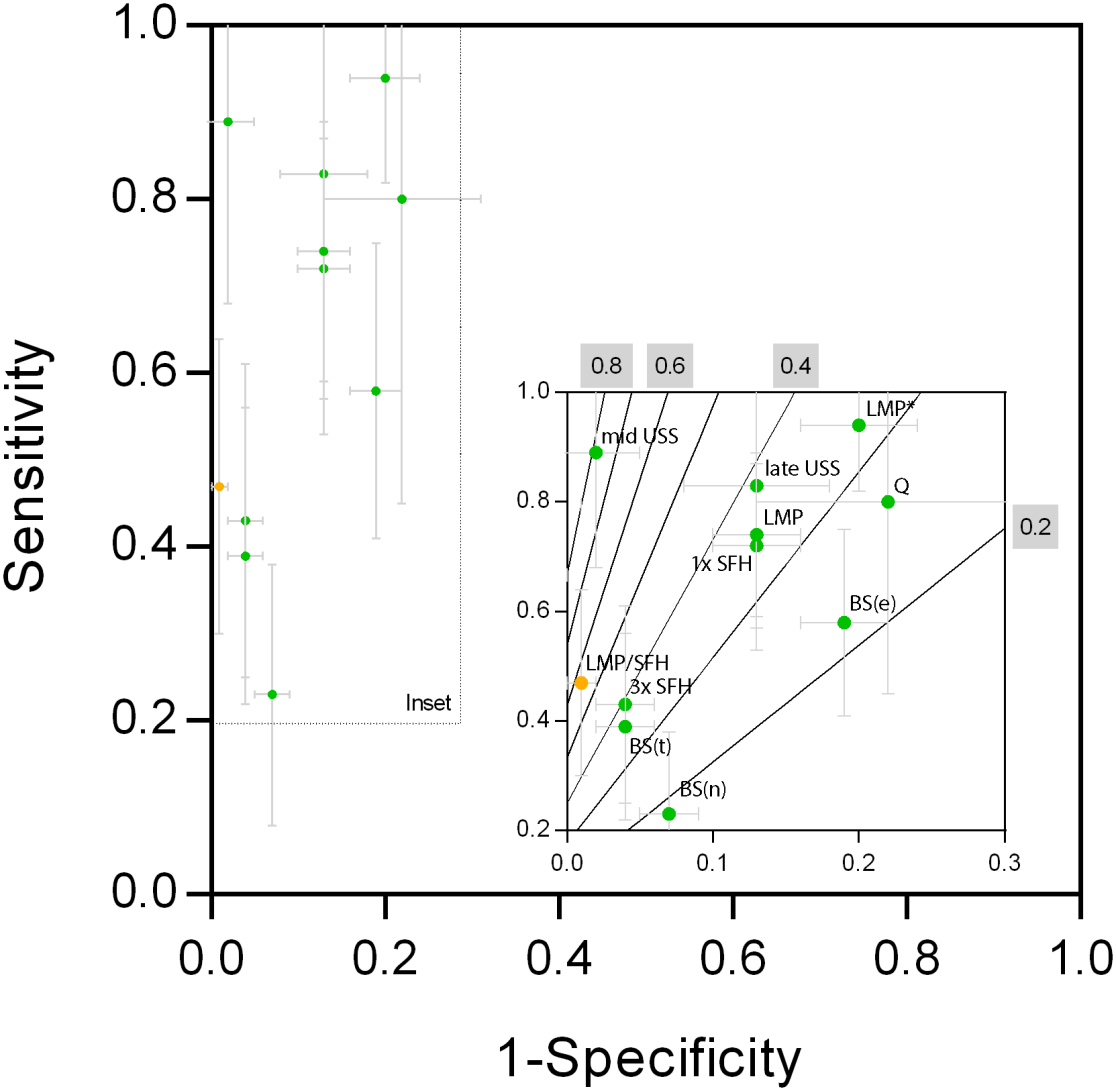
Receiver Operating Characteristic Space for Mid- and Late-Pregnancy USS and Clinical Estimators to Predict PTB. Note that the insets are magnifications of the regions of interest (outlined by the dotted lines). The solid gray lines with gray numbering are the F-measure isolines in the receiver operating characteristic space. BS(e): external BS; BS(n): neuromuscular BS; BS(t): total BS; LMP: last menstrual period; mid-scan: mid-pregnancy USS; late-scan: late-pregnancy USS; 1x SFH: linear SFH model; 3x SFH: sequential SFH model; LMP/SFH: LMP/SFH model; LMP*: corrected LMP according to PNG guidelines.

According to reference ultrasound 5.2% of neonates were preterm. The positive trend between averages and differences when comparing methods pairwise, which was observed for most of the clinical estimators, resulted in numerous false positive PTB predictions for most methods, specifically BS, LMP, late-pregnancy scans, SFH linear model, Quickening and LMP*. Table 4 summarises the performance of the methods to predict PTB in terms of sensitivity, specificity, predictive values, accuracy and F-measures. Fig 4 provides a graphical representation of the methods’ positioning in the receiver operating characteristic space including F-measure isolines.

**Table 4:** Sensitivity and Specificity of Clinical and Late Ultrasound Estimates of Gestational Age against Gold-Standard Ultrasound to Predict Preterm Birth in Comparison to the Reference Method (n = 688 with 5.2% (36) Diagnosed Preterm Birth).

Method (total number)	% (n)	Sensitivity	Specificity	PPV	NPV	Accuracy	F-measure
BS(e)^a^ (n = 623)	21.3	0.58	0.81	0.14	0.97	0.79	0.23
	(133)	(±0.17)	(±0.03)	(±0.06)	(±0.01)	(±0.04)	
BS(n)^a^ (n = 623)	8.2	0.23	0.93	0.14	0.96	0.89	0.17
	(51)	(±0.15)	(±0.02)	(±0.09)	(±0.02)	(±0.03)	
BS(t)^a^ (n = 623)	9.3	0.39	0.92	0.21	0.97	0.90	0.27
	(58)	(±0.17)	(±0.02)	(±0.10)	(±0.01)	(±0.03)	
LMP (n = 672)	15.9	0.74	0.87	0.23	0.98	0.86	0.35
	(107)	(±0.15)	(±0.03)	(±0.08)	(±0.01)	(±0.03)	
Scan between 25 & 29 GW (n = 163)	8.0	0.89	0.97	0.62	0.99	0.96	0.72
	(13)	(±0.21)	(±0.03)	(±0.26)	(±0.01)	(±0.03)	
Scan between 30 & 35 GW (n = 156)	15.4	0.83	0.87	0.21	0.99	0.87	0.34
	(24)	(±0.30)	(±0.05)	(±0.16)	(±0.01)	(±0.06)	
SFH (linear model, n = 688l)	16.4	0.72	0.87	0.23	0.98	0.86	0.35
	(113)	(±0.15)	(±0.03)	(±0.08)	(±0.01)	(±0.03)	
SFH (sequential model, n = 502l)	6.0	0.43	0.96	0.40	0.97	0.93	0.41
	(30)	(±0.18)	(±0.02)	(±0.18)	(±0.02)	(±0.02)	
Quickening (n = 84)	25.0	0.80	0.78	0.19	0.98	0.79	0.31
	(21)	(±0.35)	(±0.09)	(±0.17)	(±0.03)	(±0.10)	
LMP*(n = 354)	23.2	0.94	0.80	0.18	1	0.81	0.30
	(82)	(±0.12)	(±0.04)	(±0.08)	(±0.01)	(±0.05)	

**Note:** GW, gestational weeks; PPV, positive predictive value; NPV, negative predictive value. Please refer to Table 1 for abbreviations of the clinical methods. Numbers in parentheses under sensitivity, specificity, PPV, NPV and accuracy are 95% confidence intervals.

^a^ 97.4% (607) obtained within 72 hours of delivery; BS(e), external Ballard Score, BS(n), neuromuscular Ballard Score; LMP, last menstrual period; LMP*, corrected last menstrual period based on PNG guidelines; SFH, symphysis-pubis fundal height.

When judging method performance to predict PTB by using F-measures, mid-pregnancy USS performed best (F-measure: 0.72) followed by the SFH (sequential model, 0.41). The order of the remaining methods by F-measure was: LMP (0.35), SFH (linear model, 0.35), late-pregnancy scan (0.34), Quickening (0.31), LMP* (0.30), total BS (0.27), external BS (0.23) and neuromuscular BS (0.17). Therefore, mid-pregnancy USS is the most useful way to predict PTB in the absence of early pregnancy USS, although an F-measure of 0.72 is only in the medium range. In the absence of ultrasound facilities, the best raw measure to predict PTB was LMP.

All clinical methods had a high negative predictive value (>0.96) to predict PTB, indicating that there is a low probability that PTB infants are misclassified as being not PTB. However, positive predictive values were generally low, and consequently false classification of non-PTB infants as being PTB occurred frequently. In the absence of ultrasound, the sequential SFH model provided the highest positive predictive value (0.4), followed by the single SFH model (0.23).

### Modelling

Multiple linear regression models were fitted on combinations of clinical measures as follows: (a) LMP and SFH (linear); (b) LMP and total BS; (c) SFH (linear) and total BS; (d) LMP, SFH (linear) and total BS; (e) LMP and SFH (sequential); (f) LMP* and total BS. In order to select the best model for predicting gestational age at delivery, 10-fold cross-validation was first carried out on each of the regression models after which model (b) was excluded due to a resulting overall mean square error of 99.8 which was much higher than that of the other models (Table 5, mean square error: 69.1–77.5). The mean square error is used to assess the fit of linear regression models to avoid overfitting. The remaining regression models were then ranked according to the F-measure to assess predictive performance in detecting PTB. The LMP/SFH model, that is model (a), performed the best (F-measure: 0.58) compared to the other models (Table 5, F-measure: 0.18–0.54). The sensitivity and specificity of the six regression models for predicting PTB are presented in Table 5.

**Table 5:** Multiple Regression Models and Performance in Predicting Gestational Age and Preterm Birth.

Regression model; total number	% (n)	MSE	Sensitivity	Specificity	PPV	NPV	Accuracy	F
(a) LMP and SFH (linear); n = 672	3.1	75.4	0.47	0.99	0.76	0.97	0.97	0.58
	(21)		(±0.17)	(±0.007)	(±0.18)	(±0.01)	(±0.01)	
(b) LMP and total BS; n = 608	0.5	99.8	0.10	1	1	0.96	0.96	0.18
	(3)		(±0.10)	(±0)	(±0)	(±0.02)	(±0.02)	
(c) SFH (linear) and total BS; n = 623	2.1	69.3	0.35	0.99	0.84	0.97	0.96	0.50
	(13)		(±0.2)	(±0.004)	(±0.20)	(±0.01)	(±0.01)	
(d) LMP, SFH (linear) and total BS; n = 608	2.3	69.1	0.40	0.99	0.86	0.97	0.97	0.54
	(14)		(±0.18)	(±0.004)	(±0.18)	(±0.01)	(±0.01)	
(e) LMP and SFH (sequential); n = 491	1.63	77.5	0.22	0.99	0.75	0.96	0.95	0.34
	(8)		(±0.16)	(±0.006)	(±0.30)	(±0.02)	(±0.02)	
(f) LMP* and total BS; n = 301	0.7	72.5	0.15	1	1	0.96	0.96	0.27
	(2)		(±0.20)	(±0)	(±0)	(±0.02)	(±0.02)	

**Note:** GW, gestational weeks; LMP, last menstrual period; LMP*, corrected last menstrual period based on PNG guidelines; SFH, symphysis-pubis fundal height; BS, Ballard Score PPV, positive predictive value; NPV, negative predictive value; F, F-measure. Numbers in parentheses under sensitivity, specificity, PPV, NPV and accuracy are 95% confidence intervals. The mean square error (MSE) assesses the fit of the regression models used to predict gestational age while sensitivity, specificity, PPV, NPV, accuracy and F-measure are measures of performance in predicting PTB.

Of the 672 women included in the LMP/SFH model, 21 (3.1%) were classified as PTB. Unlike the clinical and ultrasound measures in Table 4, the LMP/SFH regression model had a negative trend in the bias as shown in the Bland-Altman plot (S3 Fig), with a mean bias of 0 and 95% limits of agreement of (-17, 17) leading to a reduced sensitivity but increased specificity to predict PTB and a better overall performance as determined by the F-measure. The intraclass correlation between GA by the reference ultrasound and that predicted by the LMP/SFH model was 0.11 (standard error = 1.26), while the corresponding concordance correlation coefficient was 0.65 (standard error = 0.02).

The LMP/SFH (linear), that is model (a), exhibited negative/positive predictive values of 0.97 and 0.76, respectively, a considerable improvement in comparison to the established methods. However, this approach requires further validation through application to other data sets. The resulting formula to calculate GA using the best performing model (a) was

GA(days)=145.04+0.07×LMP+0.40×SFH (2)

A figure with predicted GA frequency as well as the Bland-Altman and correlation plots can be found in the Supporting Material (S3 Fig).

## Discussion

This is the first published study to comprehensively assess a range of established methods to estimate GA for agreement with early-pregnancy fetal biometry in a cohort of pregnant women from rural PNG. On average, estimators predicted GA to within one week of the USS reference. However, methods differed greatly in their capability to predict PTB, owing to the fact that the bias in the agreement was subject to significant variation with GA: for lower average GA the bias was generally negative, meaning that the clinical estimator further underestimated GA, thereby decreasing the sensitivity and positive predictive value for PTB. Although some methods performed better than others, their performance to detect PTB is inadequate. However, most methods had high specificity and negative predictive value, and can be still be used to exclude PTB.

We show that mid-pregnancy USS is by far the best available alternative to detect and rule out PTB (sensitivity 0.89, specificity 0.97, F-measure 0.72), suggesting that fetal biometry remains a reasonable option to estimate GA should scanning facilities be available and women first present between 24 and 30 gestational weeks. However, such mid-pregnancy scans inevitably overestimate PTB due to early fetal growth restriction. Non-ultrasound estimators of GA with the best diagnostic performance included the sequential SFH approach by White *et al*. and LMP (F-measures of 0.41 and 0.35, respectively) [13]. These methods may be used when ultrasound is unavailable, but their performance to correctly diagnose PTB is suboptimal. We only collected a maximum of three fundal height measurements (instead of an average of nine in the original study by White et al), which may explain why the sequential SFH model performed less convincingly in our study. Performance may improve when more SFH measurements are included, which would require an increase in the number of antenatal visits: at present most women in PNG will attend four times at most. Estimating GA from LMP requires good maternal recall of dates and cycle characteristics, which may be a function of literacy (although this did not appear to be an important explanatory factor in this cohort—data not shown) [29]. More importantly, health workers are required to enquire appropriately about LMP [30]: the strong tendency of LMP to overestimate PTB may be due to women reporting (and health workers establishing) the first missed period, rather than LMP. Other studies, such as the one by Rosenberg and colleagues in Bangladesh have found that LMP is a reasonably reliable predictor of PTB [32]. When we evaluated LMP correction by SFH and quickening for 20–35 cm SFH at enrolment, as recommended by PNG guidelines, the predictive capability of the composite for PTB did not improve.

BS did not perform well for PTB prediction, although it may retain some utility for ruling out PTB. There was no difference in bias and levels of agreement for the total BS measured within 12 hr of birth and those measured later (mean bias: 6 vs. 4 days respectively; 95% CI: 34 days, for both). However, when stratifying measured GA and bias according to assessor (n = 27), there were significant differences in estimates of some health workers (S4 Fig). This suggests that inter-assessor differences may partly explain the poor performance of BS in this study, despite extensive training provided as part of the parent trial. Previous research from PNG indicated that the Dubowitz score may be of use [34]: however, 95% confidence intervals for GA predictions were wider (±3.6 weeks) compared to the original study (± 2 weeks) and similar to those we observed [15,34]. The usefulness of the BS was also shown to be limited in other low-income settings [7,35], although this is not a unanimous finding [32,36]. There is now increasing evidence to suggest that postnatal maturational assessments have a limited role for GA estimation in developing country settings and should not be used exclusively when aiming to evaluate causes of low birthweight [37].

In addition to evaluating established methods of GA estimation, we assessed the performance of a range of linear combinations of GA estimators. The precision of clinical estimators of GA to predict PTB is improved when used in combination, and use of estimates derived from such regression models may be preferable, for example, over sequential SFH and LMP alone. The model combining LMP and linear SFH provided the best estimates for PTB (using F-measure as the indicator of overall performance) and it performed better than the sequential SFH model and LMP, but not mid-pregnancy biometry. However, the model needs to be validated on other datasets in order to assess its robustness and potential clinical usefulness. For research purposes, our approach may be applied when datasets are incomplete and fetal biometric measurements need to be estimated for a fraction of study participants. As it stands however, the role of this model with regards to accurately detecting PTB is limited (sensitivity 0.47), yet may be useful for the exclusion of PTB cases (specificity 0.99).

The present study is subject to substantial limitations. Firstly, only a small number of pregnancies (3%) could be dated by first trimester ultrasound as a result of the high prevalence of late presentation to antenatal clinic in this area of PNG [21,26], and therefore reference ultrasound dating was extended to include biometric measurements taken up until 24 gestational weeks. Although this is a valid alternative and accepted practice, error margins inevitably increase with advancing GA [6,38]. In addition, fetal growth restriction in early pregnancy could lead to underestimation of GA, and hence overestimation of PTB [39]: some women in the cohort were parasitaemic and anaemic at enrolment, which may have affected the accuracy of ultrasound pregnancy dating [40]. Secondly, we used dating standards largely derived from a Caucasian population [6]. The role of ethnicity in early fetal growth is subject of ongoing debate [38]; in the absence of locally derived dating standards, use of a frequently used dating standard was the best available alternative. Thirdly, due to lack of resources we were unable to perform in-depth intra- or inter-observer variation analyses. However, all clinical staff had formal training and additionally underwent biannual refresher training. We believe that the results of our study are, at a minimum reflective of, if not better than, the realities of clinical practice in most rural areas of PNG. Although the cohort size of 688 women is considerable, unavailability of complete data for some estimates (especially quickening) limited the number of data points available for some analyses. Lastly, recruitment criteria of the parent trial (e.g., SFH <26 cm) may affect generalisability of our findings to the wider population of pregnant women in rural PNG, given late presentation to antenatal clinic is common [26].

In conclusion, clinical methods, in particular BS, were of limited use in assessing PTB in PNG. LMP retains some clinical utility and estimates based on LMP may improve with increasing literacy and further training of health workers. Mid-pregnancy fetal biometry is useful, but confounded by early fetal growth restriction. The LMP/SFH regression model developed in the present study may be applied clinically and/or to data sets lacking reliable estimates of GA, but this approach needs further validation. Our findings suggest that in order to accurately determine GA at delivery in low resource settings (whether for clinical or research purposes) we are left with two principal options: to increase the availability of obstetric ultrasound and encourage early presentation; or to develop new, simple, measures of GA, a need that has been recently identified as target area of research [41]. Antenatal ultrasound was found acceptable in other low and middle-income countries contexts (not formally assessed in our cohort) [9], and high-quality scans can be performed by locally-trained health workers [8]. This indicates that the careful and culturally appropriate introduction of ultrasound may be the way forward; whether this goes beyond estimating GA and results in improved care and pregnancy outcomes in such settings remains unclear [42].

## Supporting Information

S1 Fig Frequency Histograms of Estimated Gestational Age (GA) at Delivery for the Assessed Clinical Estimators. A) BS (external); B) BS (neuromuscular); C) BS (total); D) LMP; E) Reference USS; F) mid-pregnancy scan; G) late pregnancy scan; H) linear SFH model; I) sequential SFH model; J) Quickening; K) LMP*. Histogram bins are in weeks (7 days). Continuous lines denote medians and dashed lines denote 5% and 95% centiles
(TIF)

S2 Fig Plots Illustrating the Correlations between the Reference Ultrasound Method and the Clinical Estimators for GA at delivery. A) BS (external); B) BS (neuromuscular); C) BS (total); D) LMP; E) mid-pregnancy scan; F) late pregnancy scan; G) linear SFH model; H) sequential SFH model; I) Quickening; J) LMP*. The continuous lines are lines of identity and dashed lines are the best fit curves of the orthogonal regression analysis. Although all measures are highly correlated with the reference method, levels of agreement and concordance are generally poor.
(TIF)

S3 Fig LMP/SFH Model Developed in the Present Study. Panel A shows a histogram of the distribution of GA estimates by the novel model. Panel B shows the Bland-Altman plot showing mean bias and confidence levels of agreement between the new model and the reference ultrasound. Panel C shows the concordance plot with the orthogonal regression line.
(TIF)

S4 Fig Ballard Scores (total BS) Stratified by Assessor. Panel A: Gestational Age by total BS; Panel B: Bias between reference early pregnancy ultrasound and total BS by assessor. Only data for assessors with more than 20 measurements is shown. The red box-and-whiskers chart on the left represents the entire study population. Bias estimates for some assessors deviated significantly from the population median (Mann-Whitney Test) indicating variable performance of the assessors.
(TIF)

S1 Table Linear regression parameters for average GA vs bias from the Bland-Altman analyses. (DOCX)
