# Supplementary figures and images for: Preterm or Not – An Evaluation of Estimates of Gestational Age in a Cohort of Women from Rural Papua New Guinea
Source: PLoS One. 2015 May 6;10(5):e0124286. doi: 10.1371/journal.pone.0124286 (PMC4422681; doi:10.1371/journal.pone.0124286)

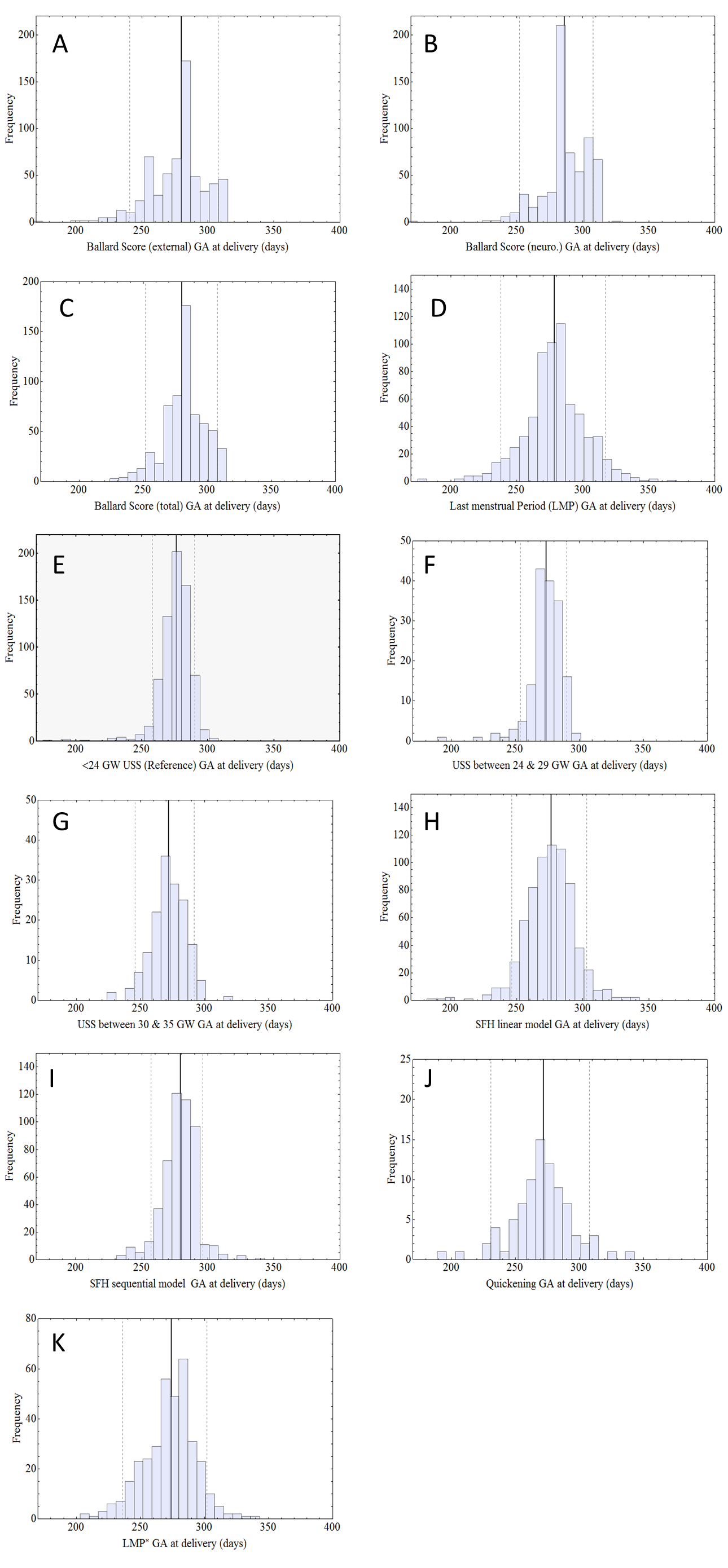

Supplement: S1 Fig — A) BS (external); B) BS (neuromuscular); C) BS (total); D) LMP; E) Reference USS; F) mid-pregnancy scan; G) late pregnancy scan; H) linear SFH model; I) sequential SFH model; J) Quickening; K) LMP*. Histogram bins are in weeks (7 days). Continuous lines denote medians and dashed lines denote 5% and 95% centiles (TIF) [file pone.0124286.s001.tif]

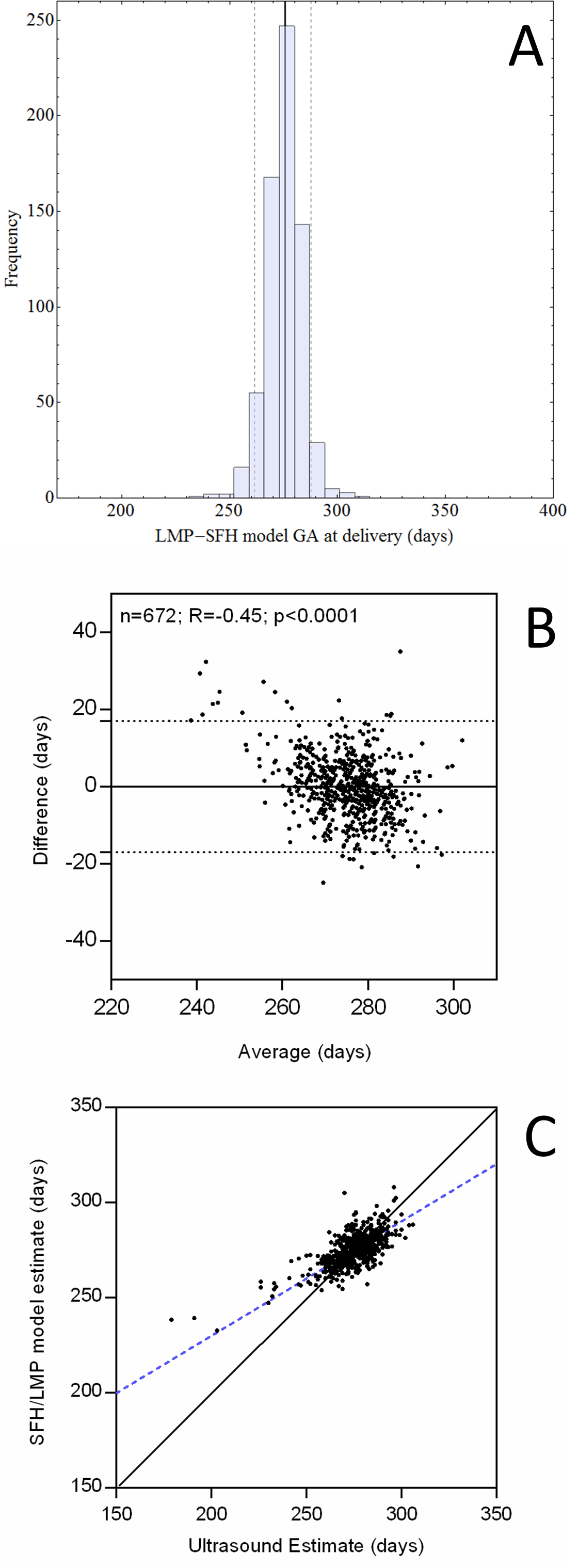

Supplement: S3 Fig — Panel A shows a histogram of the distribution of GA estimates by the novel model. Panel B shows the Bland-Altman plot showing mean bias and confidence levels of agreement between the new model and the reference ultrasound. Panel C shows the concordance plot with the orthogonal regression line. (TIF) [file pone.0124286.s003.tif]

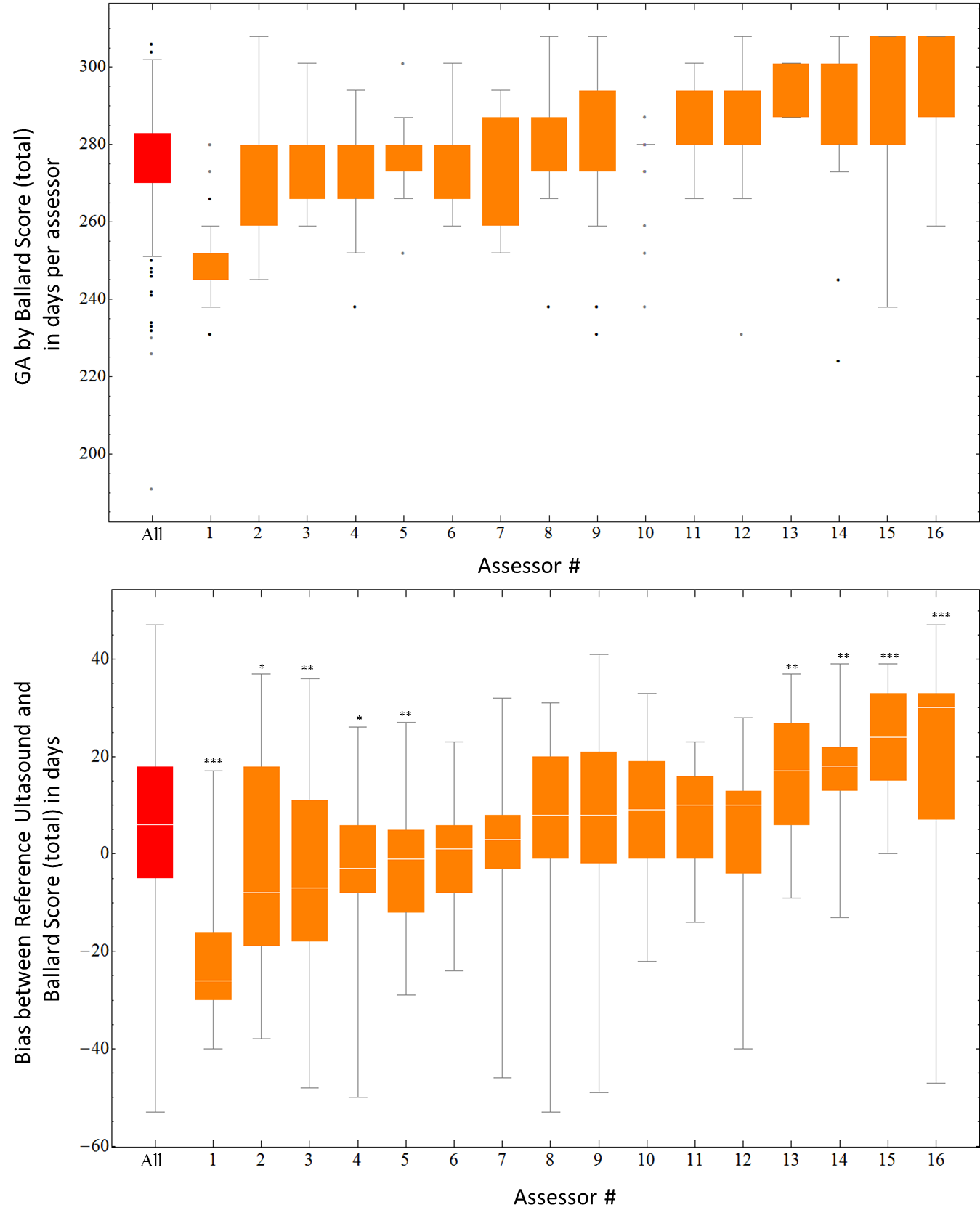

Supplement: S4 Fig — Panel A: Gestational Age by total BS; Panel B: Bias between reference early pregnancy ultrasound and total BS by assessor. Only data for assessors with more than 20 measurements is shown. The red box-and-whiskers chart on the left represents the entire study population. Bias estimates for some assessors deviated significantly from the population median (Mann-Whitney Test) indicating variable performance of the assessors. (TIF) [file pone.0124286.s004.tif]
