# Supplementary material for: Preterm or Not – An Evaluation of Estimates of Gestational Age in a Cohort of Women from Rural Papua New Guinea
Source: PLoS One. 2015 May 6;10(5):e0124286. doi: 10.1371/journal.pone.0124286 (PMC4422681; doi:10.1371/journal.pone.0124286)
Supplement: S1 Table — (DOCX) [file pone.0124286.s005.docx]

**S1 Table. Linear regression parameters for average GA vs bias from the Bland and Altman analyses.**

| Method | Slope (95% CI) | Intercept (95% CI) | R^2^ |
| --- | --- | --- | --- |
| BS(e) | 0.92 | -255.1 | 0.33 |
|  | (0.82 to 1.03) | (-283.8 to -226.4) |  |
| BS(n) | 0.66 | -175.9 | 0.15 |
|  | (0.54 to 0.79) | (-210.2 to -141.6) |  |
| BS(t) | 0.56 | -148.9 | 0.12 |
|  | (0.44 to 0.67) | (-181.1 to -116.8) |  |
| LMP | 0.85 | -231.5 | 0.56 |
|  | (0.77 to 0.93) | (-253.1 to -209.9) |  |
| Scan between 24 & 29 GW | 0.1 | -29.8 | 0.26 |
|  | (0.02 to 0.18) | (-52.7 to -6.9) |  |
| Scan between 30 & 35 GW | 0.42 | -119.6 | 0.03 |
|  | (0.31 to 0.53) | (-150.2 to -89.0) |  |
| SFH- single measurement | 0.46 | -126.6 | 0.24 |
|  | (0.40 to 0.52) | (-143.3 to -109.8) |  |
| SFH – three measurements | 0.24 | -61.6 | 0.05 |
|  | (0.14 to 0.33) | (-87.6 to -35.5) |  |
| Quickening | 0.94 | -261.2 | 0.48 |
|  | (0.72 to 1.15) | (-319.9 to -202.4) |  |
| LMP* | 1.00 | -285.1 | 0.27 |
|  | (0.86-1.21) | (-333.2 to -237.1) |  |

**Note:** GW, gestational weeks; LMP, last menstrual period; SFH, symphysis-pubis fundal height; CI, confidence interval.
